# Follistatin-Like 3 Correlates With Lymph Node Metastasis and Serves as a Biomarker of Extracellular Matrix Remodeling in Colorectal Cancer

**DOI:** 10.3389/fimmu.2021.717505

**Published:** 2021-07-16

**Authors:** Chao Yang, Fengyu Cao, Shuoyang Huang, Yongbin Zheng

**Affiliations:** Department of Gastrointestinal Surgery, Renmin Hospital of Wuhan University, Wuhan, China

**Keywords:** colorectal cancer, lymph node metastasis, extracellular matrix, tumor microenvironment, follistatin-like 3

## Abstract

**Background:**

As a heterogeneous disease, colorectal cancer (CRC) presents a great challenge to individualized treatment due to its lymph node metastasis (LNM). Existing studies have shown that immune and stromal components in extracellular matrix (ECM) act as important part in tumorigenicity and progression, while their roles in LNM have not been fully elucidated. Here, crucial ECM-related genes responsible for LNM in CRC were selected by multi-omics analysis.

**Methods:**

Firstly, we characterized the immune infiltration landscape of CRC samples from The Cancer Genome Atlas (TCGA) and the Gene Expression Omnibus (GEO) databases by using ssGSEA algorithm. The CRC patients were divided into several immune subgroups by hierarchical clustering analyses. Then, differential genes were identified among immune subgroups and CRC *vs.* normal tissues in TCGA and GEO GSE39582 cohorts, respectively. Next, weighted correlation network analysis (WGCNA) was employed to construct a co-expression network to find LNM-related modules and hub genes. Subsequently, we evaluated the clinical value of hub gene in prognostic prediction and chemotherapy/immunotherapy. Besides, the protein level of key gene was verified in an external cohort from our center. Finally, we explored the underlying mechanism of FSTL3-mediated LNM by Gene function annotation and correlation analysis.

**Results:**

Two immune subgroups, namely Immunity_High and Immunity_Low, were defined among the two CRC cohorts using ssGSEA algorithm, respectively. Based on the two immune subgroups, 2,635 overlapping differentially expressed genes were obtained from two cohorts, which were sequentially subjected to WGCNA and univariate Cox regression analysis. Ultimately, *FSTL3* was selected as the key gene. Here, we first confirmed that overexpression of *FSTL3* correlated with LNM and worse prognosis in CRC and was verified at the protein level in the external validation cohort. Moreover, FSTL3 expression showed strongly positive correlation with immune and stromal components in ECM. We furthermore found that FSTL3 may accelerate LNM through the formation of inhibitory immune microenvironment *via* promoting macrophage and fibroblast polarization and T cell exhaustion. Interestingly, high FSTL3 expression is linked to chemoresistance, but immunotherapy-sensitive.

**Conclusion:**

FSTL3 is identified as a biomarker for ECM remodeling and worse clinical outcomes for the first time in CRC and is also a potential immunotherapeutic target to block LNM for CRC.

## Introduction

Colorectal cancer (CRC), the third most common gastrointestinal malignant disease around the world, ranks second in terms of cancer-related mortality (1). Lymph nodes are common metastatic site of major types of human malignancies, including CRC. Lymph node metastasis (LNM) is a crucial event of tumor cell dissemination in that the CRC cells in the primary site spread through the lymphatic vessels to the adjacent lymph nodes (2). Usually, advanced CRC is characterized by metastases to the regional lymph nodes or distant organs, which always leads to worse prognosis. The incidence of LNM in all of CRC patients has been reported ranging from 40.5 to 49.7% (3–5). Meanwhile, LNM also is one of the most important prognostic risk factor and often predicts a poor outcome. Studies have shown that the 5-year overall survival (OS) rate is as high as 56.7 to 94.8% in CRC patients without LNM, but only 16.7 to 48% in patients with LNM-positive (6–8). Moreover, a certain number of LNM represent a key risk factor strongly associated with distant metastasis and local recurrence in CRC (9, 10). Therefore, it is necessary to identify relevant genes or elucidate potential molecular mechanisms of LNM in order to develop effective prevention and treatment strategies for CRC.

In recent years, the interaction between the tumor microenvironment (TME) and tumors has become an important aspect of tumor biology research, because it is closely related to the exploration of tumor pathogenesis and the sensitivity of immunotherapy (11, 12). The success of immunotherapy in several cancer types highlights the vital role of TME. However, as a heterogeneous disease, CRC presents a great challenge to individualized treatment due to its diversity of phenotypes and dismal prognosis. A growing body of evidence shows that tumor cells acquire stronger ability to invade and metastasize through intricate bidirectional dynamical tumor–stromal interactions in CRC (13–15). It was reported that B cells, as the major tumor infiltrating immune cells (TIICs) in TME, are able to promote lymphangiogenesis *via* targeting HSPA4 and VEGF-A, suggesting that immune components have a vital role in LNM (16). Other non-tumor cells in TME, including macrophages, fibroblasts, and neutrophils, have also been reported to act a pivotal role in progression and migration of CRC (17–19). On the other side, in TME, the composition and function of TIICs can vary slightly according to tumor progression and host immune status (20). TIICs are closely related to clinicopathological characteristics and prognosis, as well as to the efficacy of immunotherapy (21, 22). In order to obtain the best outcome of immunotherapy, it is essential to identify immune-related genes in tumor-specific phenotypes and to investigate the possible mechanisms of their functions. Hence, this study aims to discern some crucial genes involved in immune microenvironment and LNM in order to obtain better treatment results for the CRC patients with LNM.

## Materials And Methods

### Study Design and Data Acquisition

**Figure 1:**
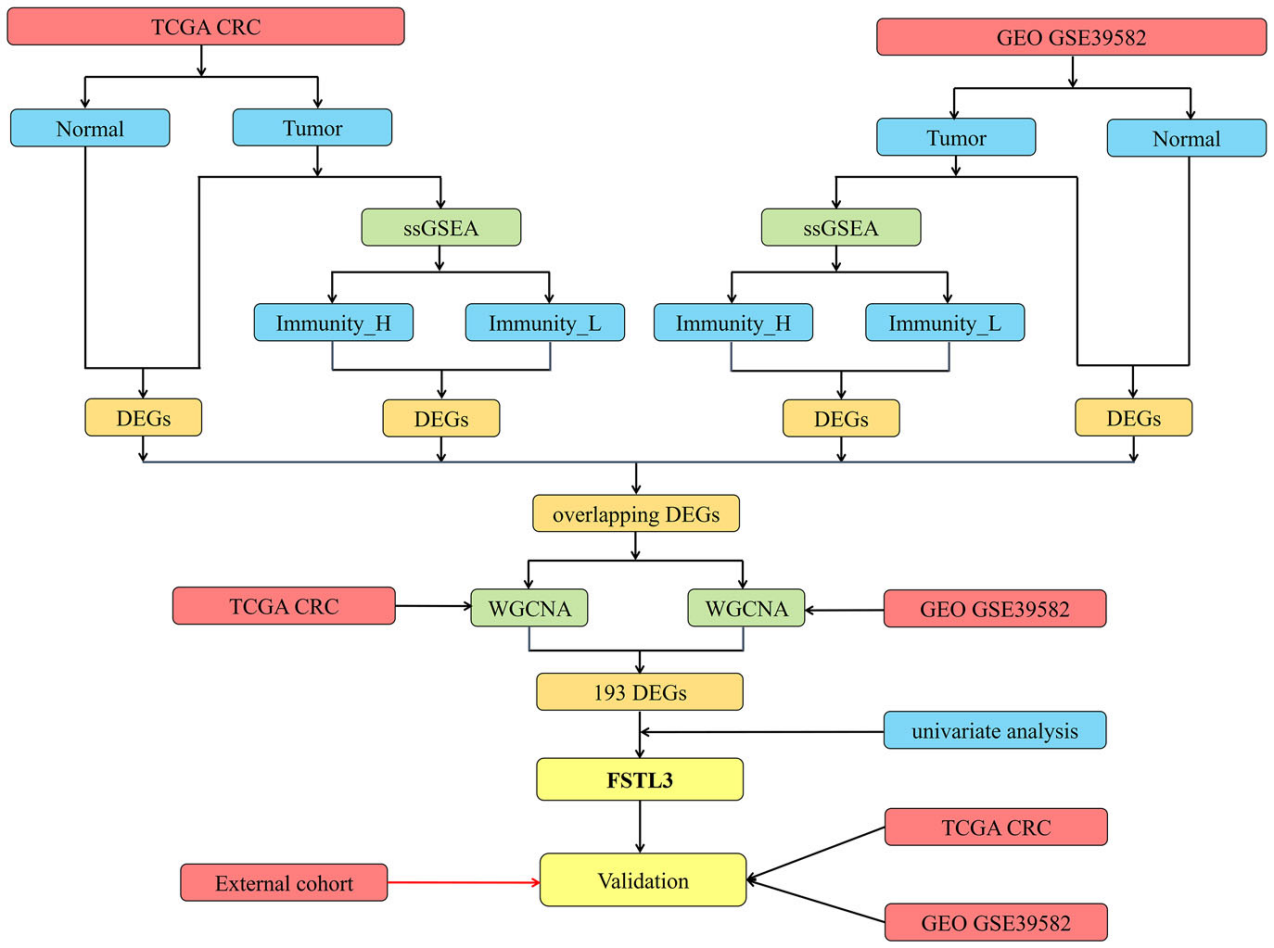
Flow chart of the present research.

Flow chart of the present research is shown in **Figure 1**. Transcriptome data (level 3 data) of RNA-seq and paired clinical data were downloaded from the TCGA CRC cohort (National Cancer Institute (NCI) and National Human Genome Research Institute (NHGRI), Bethesda, Maryland, the USA, Data Release 25.0—July 22, 2020, https://portal.gdc.cancer.gov/repository). The RNA-seq transcriptome data (FPKM) were annotated using the human General Transfer Format (hunman.gtf) from the Ensembl database (https://www.ensembl.org/) with the Strawberry Perl software (*version 5.28.2.1*, https://strawberryperl.com/).

Gene expression microarray data and clinical information of GEO GSE39582 were downloaded as a series of matrix files (23, 24). Probe IDs were matched to gene symbols using the GPL570 platform (Affymetrix Human Genome U133 Plus 2.0 Array). The mean expression value of the probes is used as the expression value for the gene in question if multiple probes were mapped to a single gene.

Criteria for study exclusion were (1): patients with unknown survival status, survival time, and N_stage; and (2) patients who died within a follow-up period of 30 days (**Table S1**).

### Specimens and Immunohistochemistry

Paraffin-embedded tissues of 68 primary CRC patients and corresponding clinical information were obtained from the Gastrointestinal Surgery Department of Renmin Hospital of Wuhan University. None of the patients received any radiotherapy or chemotherapy before the operation. The tissue specimens were collected from January 2016 to December 2017, and all patients have signed a prior informed consent. The last follow-up time was January 9, 2021. This study was approved by the Medical Ethics Committee of the Renmin Hospital of Wuhan University.

IHC staining of paraffin-embedded tissues with antibody against FSTL3 (1:25; Absin, abs101405, Shanghai, China) was performed according to standard procedures as previously described (25), and scores were recorded. The expression level of FSTL3 is evaluated according to the total histochemical score, which is calculated as the product of staining intensity and the positive cell proportion. The staining intensity is recorded as 0 (negative), 1 (weak), 2 (moderate), and 3 (strong). The positive cell proportion is scored as 0, 1, 2, 3, and 4, referring to 0, <25, 26–50, 51–75, and 76–100%.

### Clustering of CRC Samples by Single-Sample Gene Set Enrichment Analysis

The 29 immune-associated gene sets, representing diverse immune cell types, cytokine/cytokine receptor, human leukocyte antigen (HLA), immune checkpoints, immune-related pathways, and functions in TME, were obtained from a previous study (**Table S2**) (26). The enrichment scores of the 29 immune-associated gene sets in each CRC sample were quantified by ssGSEA algorithm using R package *GSVA* and *GSEABase* (27). Based on the enrichment score, the CRC patients in the TCGA and GEO GSE39582 cohorts were clustered into different immune subgroups by using R package *sparcl* (https://CRAN.R-project.org/package=sparcl).

### Assessment of the Effectiveness of Immune Clustering

The Immune/StromalScore, ESTIMATEScore, and TumorPurity were calculated by ESTIMATE algorithm in the TCGA and GEO cohorts to estimate the level of immune and stromal cells infiltrating in CRC (28). The relative proportion of 22 immune cells in CRC patients was calculated by the CIBERSORT deconvolution algorithm (29).

### Identification Differentially Expressed Genes

The R package *limma* was used to identify the DEGs among different immune subgroups, and False Discovery Rates (FDRs) < 0.05 were considered as selection criteria (30). The intersection DEGs were yielded using Venn analysis and visualized by R package *VennDiagram* (31). Firstly, we picked up overlapping differential genes between Immunity_H and Immunity_L in the TCGA and GSE39582 cohorts. Meanwhile, we acquired common differential genes between CRC and normal tissues in the TCGA and GSE39582 cohorts. Finally, we regarded the intersection of the above-mentioned results as the ultimate gene set for subsequent analyses.

### Construction of a Co-Expression Network and Recognition of the Modules Related to LNM

In this study, weighted correlation network analysis (WGCNA) algorithm is employed to construct a co-expression network to find modules associated with LNM using R package *WGCNA* (32). All of intersection genes are included into this co-expression network. Pearson correlation matrix is constructed for all gene pairs, and unsupervised hierarchical clustering analysis is performed on the basis of topological overlap matrix. Then the modules with different power values are analyzed for scale independence and average connectivity to obtain the soft threshold parameters. The optimal power value is automatically captured by the software. In this research, we set the minimal module size as 2 and the scale-independent value as 0.9 to identify the key modules correlated with LNM. Significant modules with LNM are determined using Pearson’s correlation test. A *P*-value less than 0.05 is accepted as a statistically significant difference. Venn diagram is used to select the intersection genes from WGCNA analysis among the TCGA and GEO cohorts.

### Key Gene Screening

Based on results of the previous steps, univariate Cox proportional hazard analysis is used to screen the genes with prognostic value. Consequently, these selection genes are considered as key genes associated with both the immune status, prognosis value and LNM.

### Statistical Analysis

Kruskal–Wallis test was used to compare gene expression in different samples. The correlation between gene expression and clinical characteristics was evaluated by Wilcoxon rank sum test or Chi-square test. The above analysis was performed in R software (version 4.0.2, https://www.r-project.org/). All statistical tests are two-tailed with a statistical significance level set at 0.05 in this study.

## Results

### Unsupervised Cluster Analysis Identified Two CRC Subtypes

**Figure 2:**
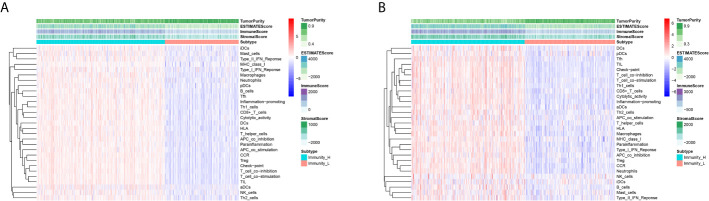
Unsupervised Cluster Analysis Identified two CRC subtypes. **(A, B)** Two immune subtypes, namely Immunity High (Immunity_H) and Immunity Low (Immunity_L), were yielded by the hierarchical clustering from the TCGA **(A)** and GEO GSE39582 **(B)** cohorts.

A total of 493 CRC samples from TCGA and 546 from GEO GSE39582 were included in this analyses. An unsupervised hierarchical clustering analysis was performed based on the ssGSEA scores of the 29 immune-associated gene sets (**Supplement Figures 1A, B** **)**. The two datasets showed similar clustering results. Two immune subgroups, namely Immunity High (Immunity_H) and Immunity Low (Immunity_L) were defined among the CRC patients from two cohorts (**Figures 2A, B** **)**.

### Correlation of CRC Immune Subtypes With Tumor Immune Microenvironment

**Figure 3:**
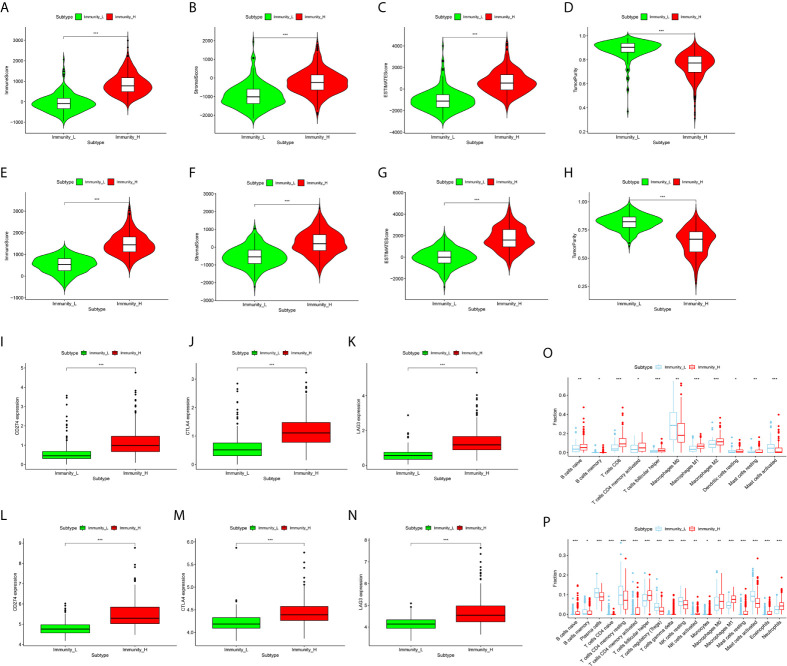
Correlation of CRC Immune Subtypes With Tumor immune Microenvironment. **(A–D)** The violin plots showed that there were significant differences in ImmuneScore **(A)**, StromalScore **(B)**, ESTIMATE Score **(C)**, and TumorPurity **(D)** between the two subtypes in the TCGA cohort (both *P* < 0.001). **(E–H)** The same results were obtained in GEO GSE39582 cohort. **(I–K)** The expression of immune checkpoints, including CD274 **(I)**, CTLA-4 **(J)**, and LAG3 **(K)**, in Immunity_H (red) are all significantly higher than that in Immunity_L (green) in TCGA cohort (both *P* < 0.001). **(L–N)** The same results were also obtained in GEO GSE39582 cohort. **(O, P)** The boxplots showed the abundance difference of each immune cell between the Immunity_H (red) and the Immunity_L (blue) in TCGA **(O)** and GEO GSE39582 **(P)** cohorts. (*P  <  0.05, **P  <  0.01, ***P < 0.001).

In order to verify the feasibility and effectiveness of the above grouping strategy, we compared the ImmuneScore, StromalScore, ESTIMATE Score, and TumorPurity between two subgroups. The Immunity_H subtype had a higher level of ImmuneScore, StromalScore and ESTIMATEScore than the Immunity_L subtype (**Figures 3A–C** **, E–G**). The changing trend of TumorPurity between two subtypes was just the opposite, indicating that Immunity_L samples contained higher number of tumor cells (**Figures 3D, H** **)**. As shown in the box plot chart, significant differences were found in the immune checkpoint (CD274, CTLA4, and LAG3) expression levels between two immune subtypes (**Figures 3I** **–N**). In addition, the two groups demonstrated marked differences in the abundance of multiple of kinds of immune cells based on the CIBERSORT algorithm (**Figures 3O, P** **)**. In summary, these results suggest that this grouping strategy is effective and can be used for subsequent study.

### Identification of Differentially Expressed Genes

**Figure 4:**
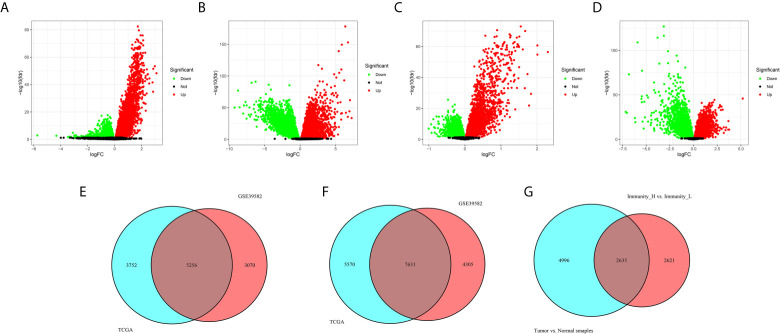
Identification differentially expressed genes. **(A, B)** The volcano plots showed the differential genes between Immunity_H and Immunity_L **(A)**, and between CRC and normal tissues **(B)** in the TCGA cohort. Each red dot represents upregulated gene, each green dot represents upregulation gene, and each black dot represents no significant difference gene. **(C, D)** The volcano plots showed the differential genes between Immunity_H and Immunity_L **(C)**, and between CRC and normal tissues **(D)** in the GSE39582 cohorts. **(E)** Overlapping differential genes between Immunity_H and Immunity_L in the TCGA and GSE39582 cohorts. **(F)** Overlapping differential genes between CRC and normal tissues in the TCGA and GSE39582 cohorts. **(G)** A total of 2,635 overlapping DEGs were obtained.

Firstly, 9,008 DEGs were identified by comparing the mRNA expression profiles across the Immunity_H and Immunity_L subtypes in TCGA cohort (**Figure 4A**). Similarly, 8,326 DEGs were identified in GEO GSE39582 cohort (**Figure 4B**). Secondly, 13,201 DEGs were obtained by comparing the mRNA expression profiles between tumor and normal samples in TCGA cohort (**Figure 4C**). Likewise, 11,936 DEGs were identified in GEO GSE39582 cohort (**Figure 4D**). There were 5,256 overlapping differential genes between Immunity_H and Immunity_L, and 7,631 overlapping differential genes between CRC and normal tissues in the TCGA and GSE39582 cohorts (**Figures 4E, F** **)**. Finally, a total of 2,635 overlapping DEGs were obtained (**Figure 4G** and **Table S3**).

### Identification of Modules Related to LNM by WGCNA

**Figure 5:**
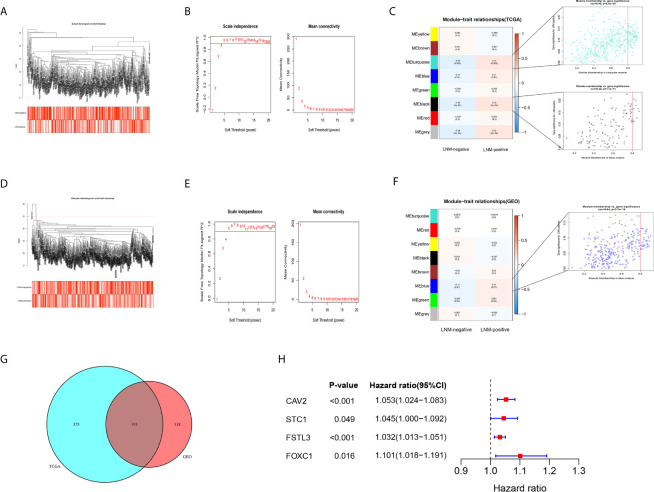
Identification of key gene by WGCNA. **(A)** The sample clustering tree of CRC samples in the TCGA cohort, and all patients have passed the cut. The color band under the sample clustering tree presents the patients with or without LNM. Red box represents LNM-positive and white box represents LNM-negative. **(B)** The scale independent value exceeds 0.9 with a low average connectivity when soft threshold power was 5. The left image shows the relationship between the soft-threshold and scale-free R^2^. The right image shows the mean connectivity for each soft-threshold. **(C)** Heatmap of the correlation between gene modules and clinical traits of CRC. The numerical value in each cell represents the correlation coefficient. Values within parentheses are *P*-values. The right images show the scatterplot of Gene Significance *vs.* Module Membership in the black and turquoise modules. **(D)** The sample clustering tree of CRC samples after removing one outlier sample (GSM972311) in the GSE39582 cohort. **(E)** The scale independent value exceeds 0.9 with a low average connectivity when soft threshold power was 5. **(F)** Heatmap of the correlation between gene modules and clinical traits of CRC. **(G)** Venn diagram showed that there were 193 overlapping genes related to LNM status in the TCGA and GSE39582 cohorts. **(H)** The forest plot showed that four genes were considered to be closely related to survival.

In this step, all 2,635 overlapping DEGs were contributed to the construction of co-expression network according to the following processes. Firstly, a sample clustering tree was drawn including all 493 CRC samples in the TCGA cohort (**Figure 5A**). Then, the scale independent value exceeded 0.9 with a low average connectivity when soft threshold power was 5 (**Figure 5B**). As shown in **Figure 5C**, WGCNA generated eight modules containing different colors after merging similar modules. Both the black, gray, and turquoise modules were closely correlated with LNM (r ≥ 0.14, *P* < 0.01). Since gray module has only two genes, we included black and turquoise modules into further analysis.

In GEO GSE39582 cohort, a sample clustering tree, including 545 CRC samples, was drawn after removing one outlier sample (GSM972311) (**Figure 5D**). The optimal power value was still 5, which was automatically selected by the software (**Figure 5E**). A total of eight modules were obtained through average hierarchical clustering. The blue module showed the highest correlation with LNM (**Figure 5F**, r = 0.11, *P* = 0.01).

### Key Gene Screening by Univariate Cox Proportional Hazards Analysis

Based on results of the WGCNA, 193 shared genes were extracted from modules closely related to LNM both in TCGA and GEO cohorts (**Figure 5G**, **Table S4**). Afterwards, univariate Cox proportional hazards analysis was carried out on those shared genes. Of the 193 overlapping genes evaluated, only four genes, namely CAV2, STC1, FSTL3, and FOXC1, were considered to be closely related to OS (**Figure 5H**, *P* < 0.05). Of them, FSTL3, related to both immune status and LNM, has previously hardly been reported in CRC. Hence, FSTL3 was selected as key gene of CRC to comprehensively investigate its value in TME and LNM in the current study.

### Overexpression of FSTL3 mRNA Correlated With Tumor Malignancy in CRC

**Figure 6:**
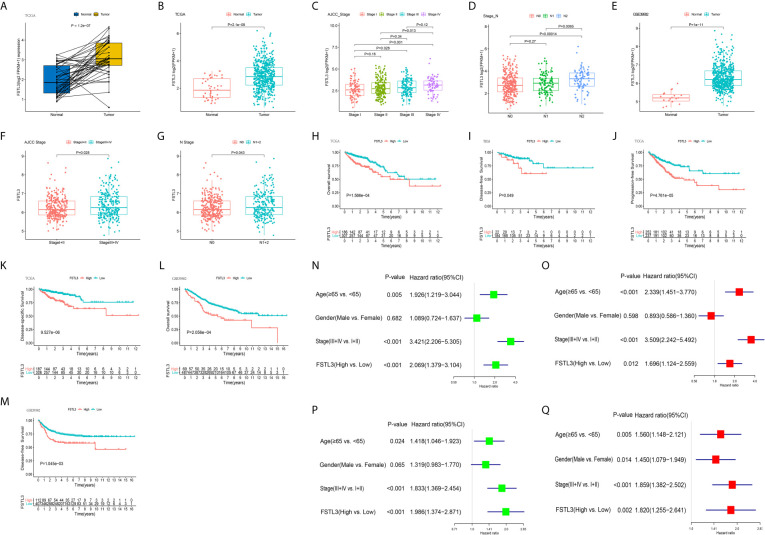
Overexpression of FSTL3 correlated with tumor malignancy in CRC. **(A)** FSTL3 mRNA expression between normal and paired tumor samples in the TCGA cohort. **(B)** FSTL3 mRNA expression between total normal and tumor samples in the TCGA cohort. **(C, D)** FSTL3 mRNA expression is in remarkable association with AJCC stage **(C)** and N stage **(D)**. **(E–G)** Similar results were obtained in GEO GSE39582 cohort. **(H–K)** Higher FSTL3 mRNA expression was notably correlated with OS **(H)**, disease-free survival **(I)**, progression free survival **(J)**, as well as disease-specific survival **(K)** in the TCGA cohort. **(L, M)** Higher FSTL3 mRNA expression is notably correlated with OS **(L)** and disease-specific survival **(M)** in the GSE39582 cohort. **(N, O)** FSTL3 is an independent unfavorable prognostic factor in TCGA cohort by univariate **(N)** and multivariate **(O)** Cox proportional hazards analysis. **(P, Q)** FSTL3 is an independent unfavorable prognostic factor in GSE39582 cohort by univariate **(P)** and multivariate **(Q)** Cox proportional hazards analysis.

The mRNA expression level of FSTL3 was markedly higher in the tumor samples than that in paired adjacent normal samples (*P* = 1.2e-07, **Figure 6A**). Meanwhile, FSTL3 mRNA expression level in cancer tissues was also higher than that in normal tissues (*P* = 2.1e-09, **Figure 6B**).

The correlation between FSTL3 expression level and the clinicopathological characteristics of CRC was further investigated. The results indicated that FSTL3 expression level was in remarkable association with AJCC stage (*P* = 0.0064, **Figure 6C**) and N stage (*P* = 0.004, **Figure 6D**). Similar results were gained from GEO GSE39582 (**Figures 6E** **–G**). Those results suggested that FSTL3 is significantly overexpressed in CRC and strongly correlated with tumor malignancy.

### High FSTL3 mRNA Level Indicates Worse Clinical Outcomes in CRC

Because our findings highlighted that FSTL3 correlated with tumor malignancy, we next went on to investigate its prognostic value in CRC. The CRC patients were divided into low- and high-expression groups based on optimal cutoff value of FSTL3 expression, which was determined using the ‘surv_cutpoint’ algorithm of the R package *survminer* (https://CRAN.R-project.org/package=survminer). The prognostic significance of FSTL3 was analyzed by Kaplan–Meier survival curves with Log-rank test. The results implied that higher FSTL3 mRNA expression was notably correlated with OS, disease-free survival (DFS), progression free survival, as well as disease-specific survival in CRC patients utilizing data from TCGA cohort (**Figures 6H–K**). Consistent results were obtained in GEO GSE39582 cohort (**Figures 6L, M**). Besides, univariate and multivariate Cox proportional hazards analysis were employed to assess whether FSTL3 could be used as an independent prognostic indicator in CRC patients. The results showed that the FSTL3 is an independent unfavorable prognostic factor both in TCGA (**Figures 6N, O** **)** and GEO GSE39582 (**Figures 6P, Q** **)** cohorts.

### FSTL3 Protein Is Remarkably Upregulated in CRC and Associated With Poor Prognosis

**Figure 7:**
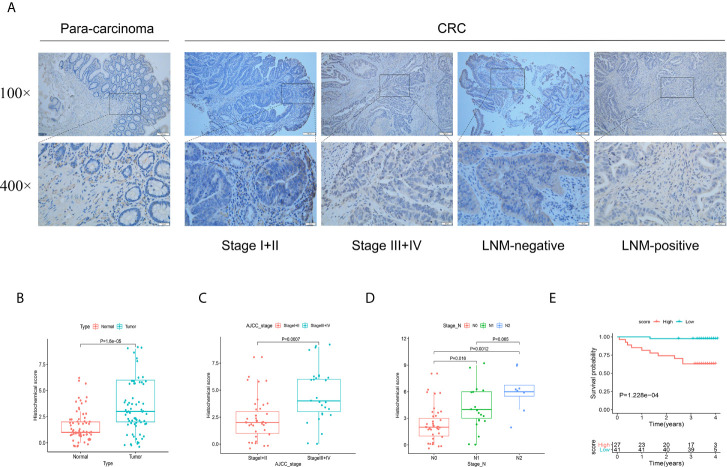
Validation the protein function of FSTL3 by immunohistochemistry in external cohort. **(A, B)** FSTL3 protein was significantly overexpressed in tumor tissues. Scale bars, 100 and 20 μm. **(C, D)** FSTL3 protein is strikingly correlated to AJCC stage (*P* = 0.0007) and LNM (*P* <  0.05). **(E)** The survival analysis showed that CRC patients with higher FSTL3 protein has shorter OS compared with lower FSTL3 protein group (*P* = 1.228e-4).

To further verify the role of FSTL3 protein in CRC progression, IHC was conducted to evaluate the FSTL3 protein expression in 68 human CRC specimens. The results showed that compared with adjacent normal tissue, FSTL3 protein was significantly overexpressed in tumor tissues (*P* = 1.6e-5, **Figures 7A, B** **)**. Further analysis revealed that the FSTL3 protein was strikingly correlated to AJCC stage (*P* = 0.0007, **Figure 7C**) and LNM (*P* <  0.05, **Figure 7D**). Additionally, we evaluated the prognostic value of FSTL3 protein expression in CRC. The cutoff value of total histochemical score in survival analysis was set according to the optimal threshold. Kaplan–Meier curve showed that CRC patients with high FSTL3 protein have shorter OS compared to those with lower FSTL3 protein (*P* = 1.228e-4, **Figure 7E**). The obtained results confirmed that the FSTL3 protein expression is related to LNM and always indicates worse clinical outcomes in CRC.

### Functional Annotation Among the High and Low FSTL3 Expression Groups

**Figure 8:**
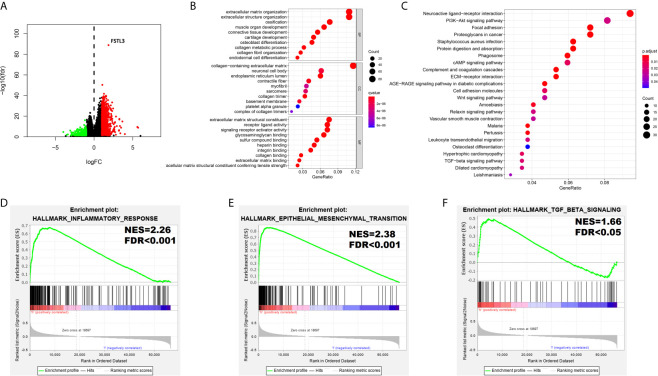
Functional Annotation among the High and Low FSTL3 Expression Groups. **(A)** The volcano plot showed that 1,331 differential genes were identified between high and low FSTL3 groups. Each red dot represents upregulation gene, each green dot represents upregulation downregulation gene, and each black dot represents no significant difference genes. **(B)** The top 10 significant terms of GO analysis. **(C)** The top 20 significant pathways of KEGG analysis. **(D–F)** The GSEA showed that inflammatory response **(D)**, epithelial–mesenchymal-transition **(E)**, and TGF-β signaling **(F)** were positively enriched in FSTL3 high-expression group.

DEGs between high and low FSTL3 groups were identified, where |log2-fold change (FC)| >1 and *P <*0.05 were set as the cutoff values. Differential expression analysis yielded a total of 779 upregulated and 552 downregulated genes in high FSTL3 expression group compared with the low FSTL3 expression group (**Figure 8A**). The top significant terms of GO and KEGG analysis shown in Bubble diagrams revealed that these DEGs were mainly associated with ECM, suggesting that FSTL3 might function as vital regulator in ECM of CRC (**Figures 8B, C** **)**.

To further identify a potential function of FSTL3, the GSEA (using the Molecular Signatures Database h.all.v6.0.symbols.gmt) was conducted (**Table S5**). The GSEA showed that substantial gene sets were positively enriched in FSTL3 high-expression group including inflammatory response (NES = 2.26, *FDR* < 0.001, **Figure 8D**), epithelial–mesenchymal-transition (EMT, NES = 2.38, *FDR* < 0.001, **Figure 8E**), and TGF-β signaling (NES = 1.66, *FDR* < 0.05, **Figure 8F**). Altogether, these results suggested that FSTL3 may promote tumor progression and LNM by regulating inflammatory response and EMT *via* TGF-β signaling.

### Correlation Between FSTL3 and TME in CRC

**Figure 9:**
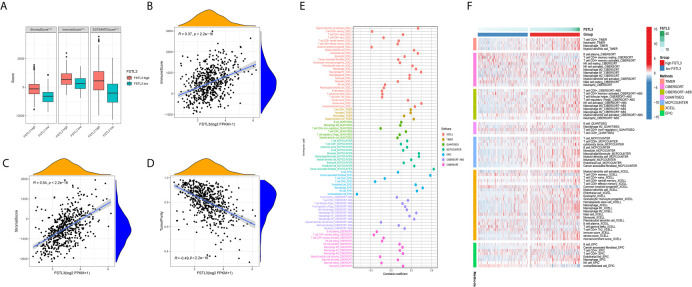
Correlation Between FSTL3 and TME in CRC. **(A)** Both the higher ImmuneScore, StromalScore, and TumorPurity were found in high FSTL3 expression group. **(B–D)** The correlation analysis showed the FSTL3 expression has a observably positive association with ImmuneScore **(B)**, StromalScore **(C)**, but negative association with TumorPurity **(D)** in CRC. **(E, F)** The relationship between FSTL3 expression and immune cell infiltration is displayed in a bubble diagram **(E)** and heatmap **(F)**, respectively. ***P < 0.001.

To better characterize the immunological role of FSTL3 plays in TME, we evaluated the relationship between FSTL3 and immune/stromal cell infiltration in CRC. Firstly, the ImmuneScore, StromalScore, and TumorPurity among the high and low FSTL3 expression groups were compared. The results showed that both the higher ImmuneScore, StromalScore, and ESTIMATE scores were found in high FSTL3 expression group (**Figure 9A**). Secondly, we found that FSTL3 expression has an observably positive association with ImmuneScore (R = 0.37, *P* < 0.001, **Figure 9B**), StromalScore (R = 0.54, *P* < 0.001, **Figure 9C**), but negative association with TumorPurity (R = −0.49, *P* < 0.001, **Figure 9D**) in CRC, which demonstrate that FSTL3 has a major influence on ECM remodeling.

The abundance of TILLs calculated by currently acknowledged methods, including TIMER, CIBERSORT-AB, XCELL, QUANTISEQ, MCPcounter, EPIC, and CIBERSORT algorithms, was downloaded from TIMER2.0 website to explore the relationship between the FSTL3 mRNA and immune infiltration status (33). The relationship between FSTL3 expression and immune cell infiltration is displayed in a bubble diagram (**Figure 9E**, **Table S6**). The analysis results showed that the FSTL3 expression was more positively associated with abundances of TIICs such as cancer-associated fibroblasts (CAFs), macrophages (M1- and M2-like), myeloid dendritic cell and CD4^+^/CD8^+^ cells, whereas it was negatively associated with B cells and NK cells. As shown in **Figure 9F**, there was obviously more immune cell infiltration in the high FSTL3 group, exhibiting a “hot” tumors phenotype (**Table S7**).

### Correlation Between FSTL3 and Immune Modulators in CRC

**Figure 10:**
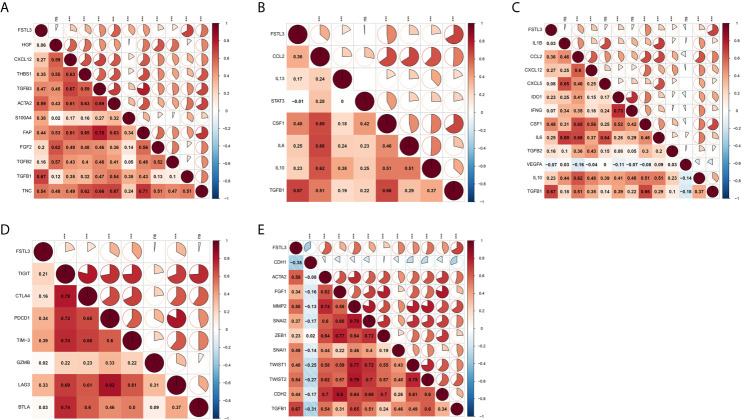
Correlation Between FSTL3 and immune modulators in CRC. **(A, B)** FSTL3 is significantly positively correlated with CAF-associated **(A)** and M2-like macrophage **(B)** genes. **(C)** The majority of immunosuppressive factors were correlated with FSTL3 positively. **(D)** FSTL3 is positively correlated with many T cell exhaustion genes in CRC. **(E)** Almost all EMT-related marker genes, except CDH1 which is biomarker of epithelial cells, are positively correlated with FSTL3 expression. ns: non-significant; *P < 0.05, **P < 0.01, ***P < 0.001.

Preliminary analyses revealed FSTL3 is more positively associated with suppressive immune cells, especially CAFs and M2-like macrophage. Therefore, we speculated that FSTL3 could be involved in inhibitory TME formation in CRC. To validate this speculation, correlation analyses were performed to explore the relationship between the FSTL3 expression and marker genes of those immune cells. CAFs, also the main cellular component in TME, are known to be involved in regulating inflammatory responses, tumor cell proliferation and migration, as well as TME remodeling by secreting matrix degrading enzymes, cytokines and growth factors (34–36). Correlation heatmap showed that FSTL3 was significantly positively correlated with CAF-associated genes and had a strong positive correlation with TGFB1 (gene encoding TGF-β) (**Figure 10A**). Tumor-associated macrophages (TAMs), often characterized by M2-like macrophages, have a variety of tumor-promoting effects in the TME (37, 38). As shown in **Figure 10B**, FSTL3 was highly positively correlated with the gene set associated with M2-like macrophage, which implicated FSTL3 plays an important role in driving M2 polarization.

It is reported that suppressive TME and T cell exhaustion are critical to for the efficacy of immunotherapy, particularly immune checkpoint inhibitors (ICIs) and chimeric antigen receptor T cell therapy (39, 40). Next, the correlation between the FSTL3 expression and immunosuppressive molecules and T cell exhaustion genes was also examined. As shown in **Figure 10C**, we observed that the majority of immunosuppressive factors were correlated with FSTL3 positively. Similarly, we observed that FSTL3 was also positively correlated with T cell exhaustion genes in CRC (**Figure 10D**). These findings suggest that FSTL3 might have participated in the formation of an immunosuppressive TME *via* promoting T cell exhaustion, either directly or indirectly.

It is well known that EMT, an important hallmark of advanced cancer, is a key step for cancer cells to acquire metastasis potential (41). As the previous GSEA analysis showed that EMT-related pathway was significantly enriched in the FSTL3 high expression group, we explored the correlation between FSTL3 expression and EMT-related marker genes here. As expected, we observed that almost all EMT-related marker genes, except CDH1, which is biomarker of epithelial cells, were positively correlated with FSTL3 expression (**Figure 10E**). Collectively, these findings offer a landscape perspective in terms of the interactive relationship among FSTL3 and TME in CRC, and FSTL3 might promote immune evasion and LNM, which functions by promoting T cell dysfunction and phenotypic transformation of M2 and CAFs, and mediating EMT.

### FSTL3 Overexpression May Contribute to the Chemotherapy Resistance

**Figure 11:**
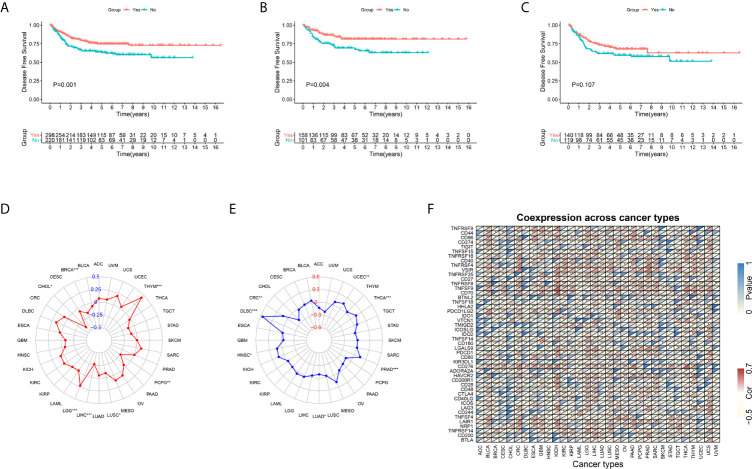
Kaplan–Meier plots and pan-cancer analysis. **(A)** CRC patients who underwent adjuvant chemotherapy had a significantly better disease-free survival. **(B)** CRC patients with lower FSTL3 could benefit from adjuvant chemotherapy. **(C)** CRC patients with high FSTL3 would gain limited benefit from adjuvant chemotherapy. **(D)** FSTL3 expression levels are correlated with TMB among multiple types of cancers, but not including CRC. **(E)** The radar plot shown that FSTL3 expression is associated with MSI among CRC. **(F)** FSTL3 expression level is significantly correlated with the expression of immune checkpoint molecules in the vast majority of cancers. *P < 0.05, **P < 0.01, ***P < 0.001.

Because complete adjuvant chemotherapy data are available in GEO GSE39582 cohort, we utilized this cohort to explore whether the FSTL3 expression level influenced the clinical outcomes of adjuvant chemotherapy in CRC. The CRC patients were separated into two groups (high and low FSTL3 expression groups) based on its median value. Overall, patients who underwent adjuvant chemotherapy had a significantly better DFS (*P* = 0.001, **Figure 11A**). The results of Kaplan–Meier survival analysis revealed that patients with lower FSTL3 could benefit from adjuvant chemotherapy (*P* = 0.004, **Figure 11B**), while high FSTL3 group showed limited benefit from chemotherapy intervention (*P* = 0.107, **Figure 11C**).

### Pan-Cancer Analysis of the FSTL3

To further confirm the guidance value of FSTL3 for individualized therapy, we conducted a pan-cancer analysis. Transcriptome RNA-seq and mutation data of pan-cancer were extracted from UCSC Xena (https://xenabrowser.net/datapages/) (42). Firstly, we evaluated the relationship between FSTL3 and TMB and MSI among multiple types of cancers, which were both regarded as independent predictors of immunotherapy efficacy (43). We found that FSTL3 expression levels were correlated with TMB among multiple types of cancers, including BRCA, CHOL, LGG, LIHC, LUSC, PCPG, and THYM, but not CRC (**Figure 11D**, **Table S8**). Patients with MSI-high are always particularly responsive to immunotherapy than those with MSI-low (44). As shown in the radar plot, FSTL3 expression was associated with MSI among CRC, DLBC, HNSC, LUAD, PRAD, THCA, and UCEC (**Figure 11E**, **Table S9**). In addition, we found that FSTL3 expression level was significantly correlated with the expression of immune checkpoint molecules in the vast majority of cancers (**Figure 11F**).

## Discussion

LNM has a decisive role in affecting the pathological stage and clinical outcomes in many human malignancies (16). Although immunotherapy has shown promising clinical results in a variety of tumors, curing CRC patients with LNM remains a significant challenge. Therefore, the development of new immunotherapy by targeting a crucial regulator in LNM and tumor progression is a feasible therapeutic strategy. The present study aims to determine the key gene which conduced to develop cancer immunotherapy to potently blocked LNM. In this study, FSTL3 was identified as a key gene associated with TME and LNM ultimately.

FSTL3, *i.e.*, follistatin-like 3, is a secreted glycoprotein that is physiologically released by adipose tissue, reproductive, glands, liver, heart, and especially placenta. Recently, significant overexpression was also found in some malignant tumors (25, 45, 46). FSLT3 expression has been shown to be significantly associated with LNM and worse outcomes in patients with non-small cell lung cancer cell and thyroid cancer (25, 45). However, the role of FSTL3 in tumor progression and cancer immunology of CRC has been rarely studied. In this research, we have comprehensively investigated the role of FSTL3 in CRC progression, clinical outcomes, and chemotherapy resistance. Importantly, we assessed the correlation of FSTL3 expression with immune and stromal components in more depth. Besides, the protein expression level of FSTL3 was verified by IHC in an external validation cohort from our center. Collectively, the findings of this study indicate that FSTL3 is a promising biomarker of ECM remodeling and immunotherapy response in CRC patients.

LNM is one of the most important prognostic markers in many types of cancers. Research has shown that tumor cells will get a stronger ability of LMN when TGF-β-induced EMT (47). Molecularly, EMT has a crucial role in driving tumor invasion and migration by promoting the expression of transcription factors and mesenchymal markers and inhibiting epithelial marker expression (48). The results of GSEA and correlation analysis showed EMT and TGF-β signaling pathway are significantly enriched in the high FSTL3 expression group. This suggests that FSTL3 may facilitate LNM *via* EMT and TGF-β signaling pathway in CRC. Certainly, additional experimental evidence is required to verify this conjecture.

Upregulation of immune checkpoint molecules is one of the main mechanism of immune evasion in many solid tumors (49, 50). In the present study, a strong correlation was observed between the FSTL3 expression and T cell exhaustion genes in CRC. Meanwhile, the results of the in-depth exploration show that among those TILs, the FSTL3 has the strongest correlation with CAFs and M2-macrophages, which are both immunosuppressive cells. Thus, it can be supposed that FSTL3 may contribute to format a tumor-promoting microenvironment by negatively regulating immunity. Consistent with this evidence, this study showed that FSTL3 was significantly correlated with PD1 and PD-L1, which are immune checkpoints. In consideration of the good therapeutic effect of Pembrolizumab and Nivolumab in CRC (51, 52), we speculated that ICIs may also be effective in CRC patients with high FSTL3 expression.

Macrophages have strong plasticity and functional heterogeneity, and they will be polarized as the local microenvironment changes. TAMs mediate tumor progression by secreting anti-inflammatory cytokines, angiogenic factors, and proteases (53, 54). In view of the plastic property of TAMs in TME, it is potential to develop a new therapy by repolarizing the M2-macrophages to become the tumoricidal M1-macrophages (55). Although platinum- and fluorouracil-based chemotherapy regimes effectively improve OS in CRC patients, some of them experience varying degrees of chemotherapy resistance phenomenon. Nevertheless, the molecular mechanisms of chemotherapy resistance in CRC remain largely unclear. Previous research has reported that CAF-derived exosomes might be involved in the enhanced chemoresistance by promoting cell stemness and EMT in CRC (56). Consistent with those findings, the present study has revealed that FSTL3 plays a vital role in EMT and contributes to the chemotherapy resistance in CRC. Interestingly, the study by Zhang et al. found that the TAMs and CAFs in CRC were able to synergistically inhibit the function of NK cells, which can kill tumor cells directly though innate immune signaling pathways (57). Hence, targeted therapy against FSTL3 may restore the body’s own anti-tumor immunity and the chemotherapy sensitivity in CRC.

In summary, in this study, FSTL3 is considered as a key gene both associated with ECM and LMN in CRC. At first, the study thoroughly evaluated the clinical and prognostic value of FSTL3 in CRC, and was verified at the protein level *in vitro*. Then, we comprehensively explored the relationship between FSTL3 expression and immune and stromal components in TME, highlighting the crucial immunological role of FSTL3 in CRC. Besides, we confirmed that FSTL3 could serve as a predictor of sensitivity to both immunotherapy and adjuvant chemotherapy in CRC. In short, FSTL3 is first reported as a biomarker of ECM remodeling and immunotherapy, and is involved in promoting LNM in CRC.

## Data Availability Statement

The original contributions presented in the study are included in the article/**Supplementary Material**. Further inquiries can be directed to the corresponding author.

## Ethics Statement

The studies involving human participants were reviewed and approved by the Medical Ethics Committee of the Renmin Hospital of Wuhan University. The patients/participants provided their written informed consent to participate in this study.

## Author Contributions

YZ and CY designed this study. CY, SH, and FC performed the data analyses and drafted the manuscript. CY and FC carried out data management and constructive discussions. All authors contributed to the article and approved the submitted version.

## Funding

The study was supported by the grants of the Wu Jieping Medical Foundation (320.2710.1855).

## Conflict of Interest

The authors declare that the research was conducted in the absence of any commercial or financial relationships that could be construed as a potential conflict of interest.
