# Supplementary figures and images for: Follistatin-Like 3 Correlates With Lymph Node Metastasis and Serves as a Biomarker of Extracellular Matrix Remodeling in Colorectal Cancer
Source: Front Immunol. 2021 Jul 16;12:717505. doi: 10.3389/fimmu.2021.717505 (PMC8322704; doi:10.3389/fimmu.2021.717505)

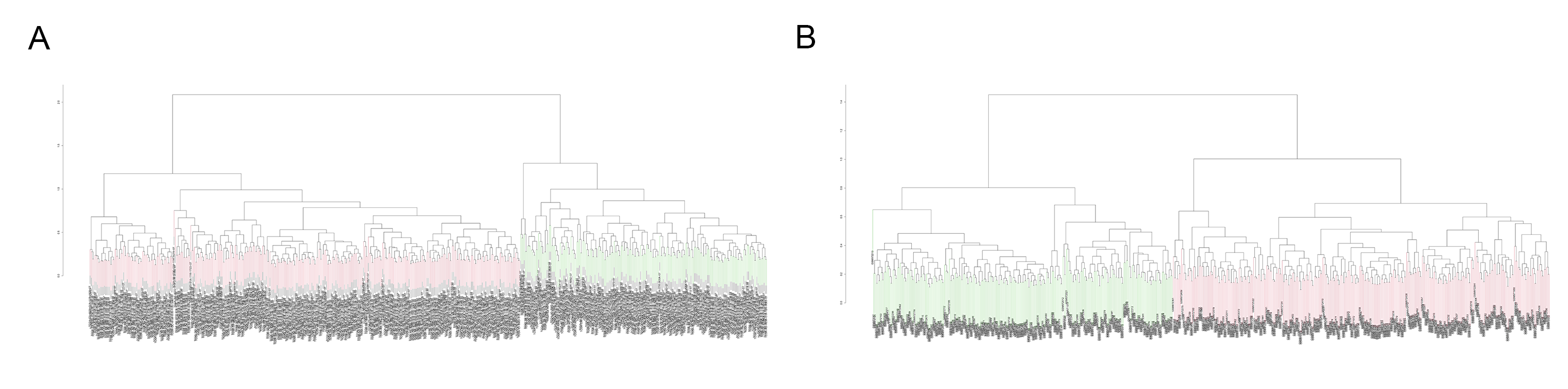

Supplement: Supplementary file 1 [file Image_1.tif]
